# Biochemical and Computational Analysis of the Substrate Specificities of Cfr and RlmN Methyltransferases

**DOI:** 10.1371/journal.pone.0145655

**Published:** 2015-12-23

**Authors:** Eleni Ntokou, Lykke Haastrup Hansen, Jacob Kongsted, Birte Vester

**Affiliations:** 1 Department of Biochemistry and Molecular Biology, University of Southern Denmark, Odense, Denmark; 2 Department of Physics, Chemistry and Pharmacy, University of Southern Denmark, Odense, Denmark; Universität Stuttgart, GERMANY

## Abstract

Cfr and RlmN methyltransferases both modify adenine 2503 in 23S rRNA (*Escherichia coli* numbering). RlmN methylates position C2 of adenine while Cfr methylates position C8, and to a lesser extent C2, conferring antibiotic resistance to peptidyl transferase inhibitors. Cfr and RlmN show high sequence homology and may be evolutionarily linked to a common ancestor. To explore their individual specificity and similarity we performed two sets of experiments. We created a homology model of Cfr and explored the C2/C8 specificity using docking and binding energy calculations on the Cfr homology model and an X-ray structure of RlmN. We used a trinucleotide as target sequence and assessed its positioning at the active site for methylation. The calculations are in accordance with different poses of the trinucleotide in the two enzymes indicating major evolutionary changes to shift the C2/C8 specificities. To explore interchangeability between Cfr and RlmN we constructed various combinations of their genes. The function of the mixed genes was investigated by RNA primer extension analysis to reveal methylation at 23S rRNA position A2503 and by MIC analysis to reveal antibiotic resistance. The catalytic site is expected to be responsible for the C2/C8 specificity and most of the combinations involve interchanging segments at this site. Almost all replacements showed no function in the primer extension assay, apart from a few that had a weak effect. Thus Cfr and RlmN appear to be much less similar than expected from their sequence similarity and common target.

## Introduction

Cfr and RlmN RNA methyltransferases are radical *S*-adenosyl-L-methionine (SAM) dependent enzymes that use a radical reaction mechanism to modify RNA by transfer of methyl groups [1, 2]. The *cfr* gene was reported in 2000 and identified on plasmid pSCFS1 from *Staphylococcus sciuri* causing resistance to florfenicol and chloramphenicol [3]. Later Cfr was found to be responsible for bacterial resistance to six classes of antibiotics binding near or at the peptidyl transferase centre (PTC) in the ribosome (phenicols, lincosamides, oxazolidinones, pleuromutilins, streptogramin A and 16-membered macrolides) [4–7]. Now the *cfr* gene is found in various bacteria and locations [8–13] but always on plasmids or in relation with transposon sequences. Bacterial strains containing Cfr are becoming a real threat due to resistance to multiple antibiotics and especially resistance to linezolid [10, 12–14]. The original parent host for the *cfr* gene has not been identified but genes coding for Cfr-like enzymes with the same functions as Cfr have been found in some bacteria [15–17]. Cfr causes resistance by methylation of C8 at 23S rRNA position A2503 [18, 19], and this is so far the only C8 methylation in natural RNA. Cfr also methylates C2 at A2503 to some extent [19]. RlmN was first reported in 2008 [20], and *rlmN* genes are apparently present in most bacteria [15]. RlmN is responsible for C2 methylation of A2503 of 23S rRNA [20, 21] and can also modify some tRNAs, at C2 of A37 [22], and possibly play a role in control of translational accuracy [23].

**Fig 1:**
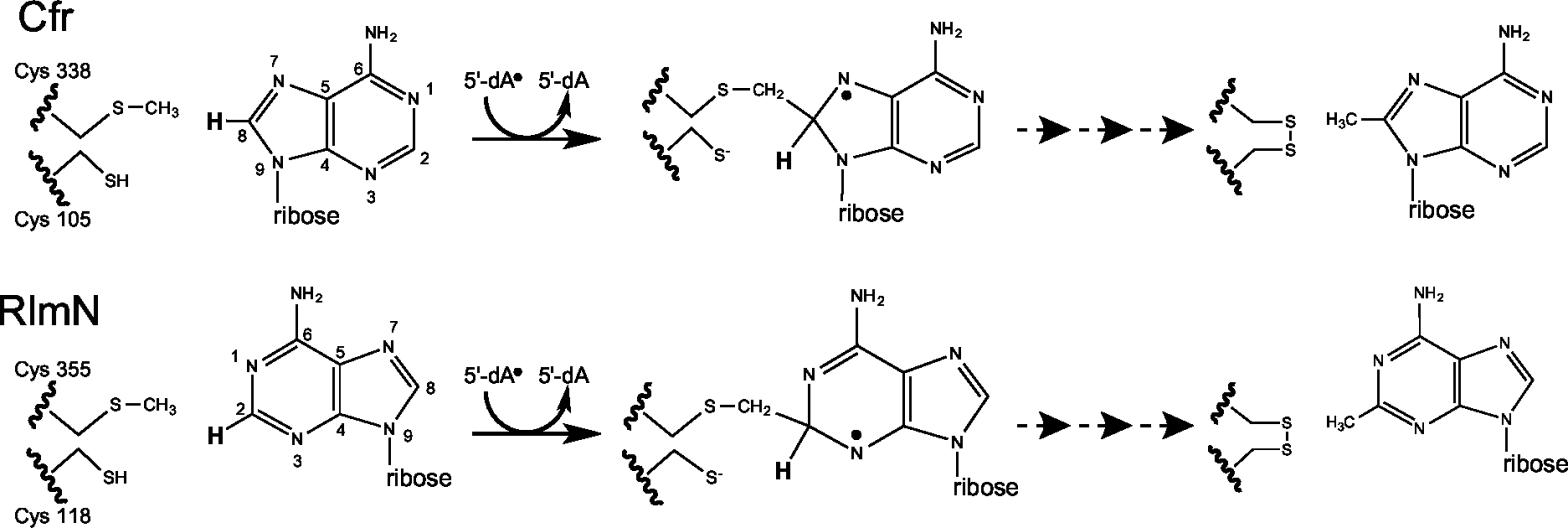
Mechanisms of action of Cfr and RlmN. A simplified version of the mechanisms of action of Cfr and RlmN proposed by Grove *et al* [1, 2] starting with the methylated Cys 338/355 (as used in our computational approach) that has been generated by attacking the activated methyl group of the first SAM. Reductive cleavage of a second SAM gives an 5’-deoxyadenosyl 5’ radical, as shown in the figure, that abstracts a hydrogen atom from the mCys338/355 group to yield a neutral, carbon-centered radical. The resulting methylene radical adds to C8/C2 of A2503 in 23S rRNA generating a protein-RNA crosslink that contains an unpaired electron (not shown). Loss of an electron and abstraction of the proton (shown in bold) from C8/C2 by a general base, results in the resolution of the covalent crosslink by disulfide bond formation that involves a second cysteine (Cys105/118).

Both Cfr and RlmN have a conserved CX_3_CX_2_C motif, indicative of radical SAM enzymes [24]. It has been shown that single mutations of each of the cysteines in the motif inactivate Cfr, suggesting that Cfr operates through a radical-based mechanism [19]. Cfr and RlmN have also been shown to consume two SAM molecules per reaction, one as a methyl donor and the other as a supporter of a radical [25]. Additionally, they bind a [4Fe-4S] cluster that works as a cofactor by supplying the essential electron for reductive cleavage of SAM [24]. The X-ray crystal structure of RlmN with the [4Fe-4S] cluster and one SAM molecule shows that the cysteine motif binds the [4Fe-4S] cluster and indicates the position of the active site [1]. Later work proposed and showed a unique methylation mechanism for RlmN and Cfr [1, 2, 26]. A simplified version of the proposed mechanism of action of both Cfr and RlmN is shown in Fig 1. The mechanism involves a transitory methylation of cysteine 338/355 (Cfr and RlmN numbering, respectively), and generation of a 5’-deoxyadenosyl 5’ radical. The methyl group is then transferred to A2503 of the 23S rRNA via a transitory crosslinking where the radical helps the cleavage of an unreactive C-H bond at A2503 [1, 26, 27].

Phylogenetic analysis by database searches of *cfr*- and *rlmN* similar genes followed by amino acid sequence alignments, point to an evolutionary relationship between the Cfr and RlmN enzymes [15, 28]. An evolutionary relationship is also supported by the fact that they recognise the same RNA target and operate via the same radical mechanism. Despite the knowledge of an RlmN X-ray structure, ligand binding and the apparent similarity of the two enzymes there is no distinguishable information to explain the different C2/C8 specificity of the enzymes.

Our objective was to investigate the specificity and relation of Cfr and RlmN using a computational and a microbiological approach. The working hypothesis was that RlmN binds the substrate in one specific configuration while Cfr was able to accept the substrate in two different configurations, methylating at either C8 or C2. Cfr was thus expected to contain a wider or more flexible binding cavity at the catalytic site than RlmN. The computational approach was used to study the structural constraints in the catalytic sites of the enzymes. First, the RlmN X-ray crystal structure [26] was used to create a Cfr structure homology model. Then the target binding capabilities of both enzymes were explored with a mononucleotide ligand followed by analysis with a trinucleotide ligand using docking and molecular dynamics (MD) simulations. The functional relationship between the Cfr and RlmN was also explored by constructing and investigating combinations of the two genes that code for the enzymes. By replacing amino acids from one enzyme with the counterpart from the other enzyme within regions of the catalytic site, we expected to identify which regions are responsible for the C2/C8 specificity. However, all results pointed to Cfr and RlmN being two distinct enzymes despite their common target, common unique mechanism of action, and sequence similarities.

## Materials and Methods

### Molecular dynamics simulations and calculation of the binding free energy

A Cfr homology model was generated based on the X-ray crystal structure of RlmN (PDB file 3RFA) [26] utilizing the Cfr sequence (GI: 34328031 / NCBI: NP_899167.1) and the I-TASSER server [29] and was prepared with the Maestro software using the protein preparation wizard in the Schrödinger Suite (Schrödinger Release 2014–1: Schrödinger Suite 2014–1 Protein Preparation Wizard; Epik version 2.7, Schrödinger, LLC, New York, NY, 2013; Impact version 6.2, Schrödinger, LLC, New York, NY, 2014; Prime version 3.5, Schrödinger, LLC, New York, NY, 2014.). Then the RlmN X-ray crystal structure, and the Cfr homology model with a target ligand were used to calculate docking scores measured in kcal mol^-1^, called Glide Scores. The docking calculations were performed using the Glide program in XP (extra precision) mode (Small-Molecule Drug Discovery Suite 2014–3: Glide v, Schrödinger, LLC, New York, NY, 2014). The Glide Score gives an estimate of how strongly a ligand binds at the binding site (the more negative the value is, the stronger the ligand binds). First, we calculated the Glide Score using AMP and then we extended the ligand to a trinucleotide (GpApU). The trinucleotide was docked in both Cfr and RlmN in four starting poses. MDs were conducted for both Cfr and RlmN with the four poses of the trinucleotide ligand, producing a total of eight trajectories with the program from the Schrödinger Release 2014–4: MacroModel, version 10.6, Schrödinger, LLC, New York, NY, 2014. The MDs were conducted with a simulation time of 2000ps, a time-step of 1fs, at 310 K (36.8°C), utilizing the OPLS 2005 force field. Only atoms within 15Å from the ligand were explicitly simulated–all atoms outside 15Å from the ligand were kept restrained using a force constant of 400 (kcal mol^-1^Å^-1^). Ten snapshots for each trajectory were obtained during the MDs, for which the Δ*G* was calculated by the MM/GBSA method [30, 31] and subsequently the <Δ*G*> per trajectory. As we did not ascertain the entropic contribution to the binding energy (TΔ*S*) of the model system we present data as <ΔΔ*G*> values using the pose with the smallest affinity average as reference point. Finally, a visual inspection of the ligand placement in the two enzymes resulting from the MD calculations was performed to evaluate the extent of structural changes.

### Bacterial strains used for plasmid construction, minimal inhibitory concentration analysis, and modification

All *E*. *coli* strains were grown in LB medium and in the presence of 100 μg ml^-1^ ampicillin for plasmid selection and maintenance. Strain *E*. *coli* TOP10 (Invitrogen) was used for transformation of ligated plasmids. The hyperpermeable *E*. *coli* AS19 strain [32] was used as a host for antibiotic susceptibility testing due to its high sensitivity to antibiotics and analysed by minimal inhibitory concentration (MIC) testing. The *E*. *coli* JW2501-1 with *rlmN* knock-out [33], was used for modification analysis by primer extension, to avoid interference from chromosome coded RlmN methylation.

### Construction of plasmids encoding mixed genes

The genes manipulated in this study contain an AvrII restriction site at their 5’ end, an XhoI restriction site at their 3’ end and a BamHI restriction site situated between regions 2 and 3. They were cloned into plasmid pBR322 [34] replacing the coding region of the *tet* gene. The BamHI site is naturally present in *cfr* but was generated in *rlmN*, resulting in an RlmN protein with mutation D198P that does not affect the function of RlmN. The genes *cfr*, *cfr1234567rrlmN* and *rlmN1234567rcfr* were purchased by Genescript and optimized to *E*. *coli* codon usage. The fragments of the genes *cfr4XrrlmN*, *cfr5XrrlmN*, *cfr6XrrlmN*, coding for the area downstream the BamHI site, were purchased by Genescript and optimized to *E*. *coli* codon usage. Using the BamHI restriction enzyme we replaced the second part of the *cfr* gene with the purchased fragments forming the genes *cfr4XrrlmN*, *cfr5XrrlmN*, *cfr6XrrlmN*. Plasmids encoding the mixed genes *cfr234567rrlmN*, *cfr1XrrlmN*, *cfr2XrrlmN*, *cfr3XrrlmN* and *cfr7XrrlmN* were constructed using overlap extension PCR with plasmids pBRCfr, pBRCfr12rRlmN and pBRCfr34567rRlmN as templates. Plasmids encoding the genes *cfrrlmN*, *cfr12rrlmN*, *cfr34567rrlmN*, *rlmNcfr*, *rlmN12rcfr*, *rlmN34567rcfr* were constructed using the restriction sites AvrII, XhoI and BamHI and creating combinations of partial fragments of the different genes. All plasmids were initially transformed into *E*. *coli* strain Top10 and then retransformed into *E*. *coli* strains AS19 and JW2501-1. All plasmid constructs were sequenced at the inserted gene to verify the identity of the cloned genes.

### Primer extension analysis to analyse modification at A2503 23S rRNA

Overnight cultures of *E*. *coli* JW2501-1 cells harbouring the plasmids were diluted and grown at 37°C for about 3 h, until reaching OD_450_ = 0.375. Then RNA was extracted with a GeneJET RNA purification kit (Thermo Scientific). Modification of ribosomal RNA was monitored by primer extension analysis [35] with AMV reverse transcriptase (Roche) using the Cy5-labeled deoxyoligonucleotide primer (5’**-**GAACAGCCATACCCTTG-3**’**), complementary to nucleotides 2,540 to 2,556 of *E*. *coli* 23S rRNA. The resulting cDNA extension products were separated on 13% polyacrylamide sequencing gels and the visualization was achieved by a fluorescence scan with Typhoon TRIO Variable Mode Imager (Amersham Bioscience). The positions of the stops were identified by referencing to dideoxynucleotide sequencing reactions on 23S rRNA, which were electrophoresed in parallel.

### Antibiotic susceptibility testing of *E*. *coli* AS19 with selected plasmids

Drug susceptibility testing was done, as described previously [36], using a microtiter plate format and measuring optical density values at 450 nm with a microtiter plate reader (Victor 3 spectrophotometer, PerkinElmer). LB medium was inoculated with single colonies and incubated overnight at 37°C. The overnight cultures were diluted to an OD_450_ = 0.01 and 100 μl of diluted culture was mixed with 100 μl of antibiotic solution in a series with 2-fold concentration steps. The tested concentration ranges were: for chloramphenicol, 0.5 to 32 μg ml^-1^; for linezolid, 2 to 128 μg ml^-1^; for tiamulin 1 to 64 μg ml^-1^. The MIC was defined as the drug concentration with no visible growth after 24 h of incubation at 37°C.

## Results and Discussion

### Theoretical calculation of target binding affinities

Cfr methylates A2503 of 23S rRNA at C8 and C2, while RlmN only performs a C2 methylation using the same mechanism of function. Thus, it is expected that certain amino acid sequence differences account for this bias. A simple way to explain this distinction is that it is caused by steric hindrance. Our initial assumption was that Cfr evolved to be able to accept the substrate in different configurations giving it the ability to methylate both C2 and C8 of the A2503. A wider and more flexible binding cavity in Cfr than RlmN at the catalytic site could allow entry and binding of adenine in two different configurations, permitting the enzyme to perform methylation at two different carbon atoms on the target adenine. To test this hypothesis we conducted a theoretical investigation employing the Molecular Mechanics/Generalized Born Surface Area (MM/GBSA) [31] [30] method to estimate binding affinities of substrates and molecular dynamics to further investigate structural effects of substrate binding.

**Fig 2:**
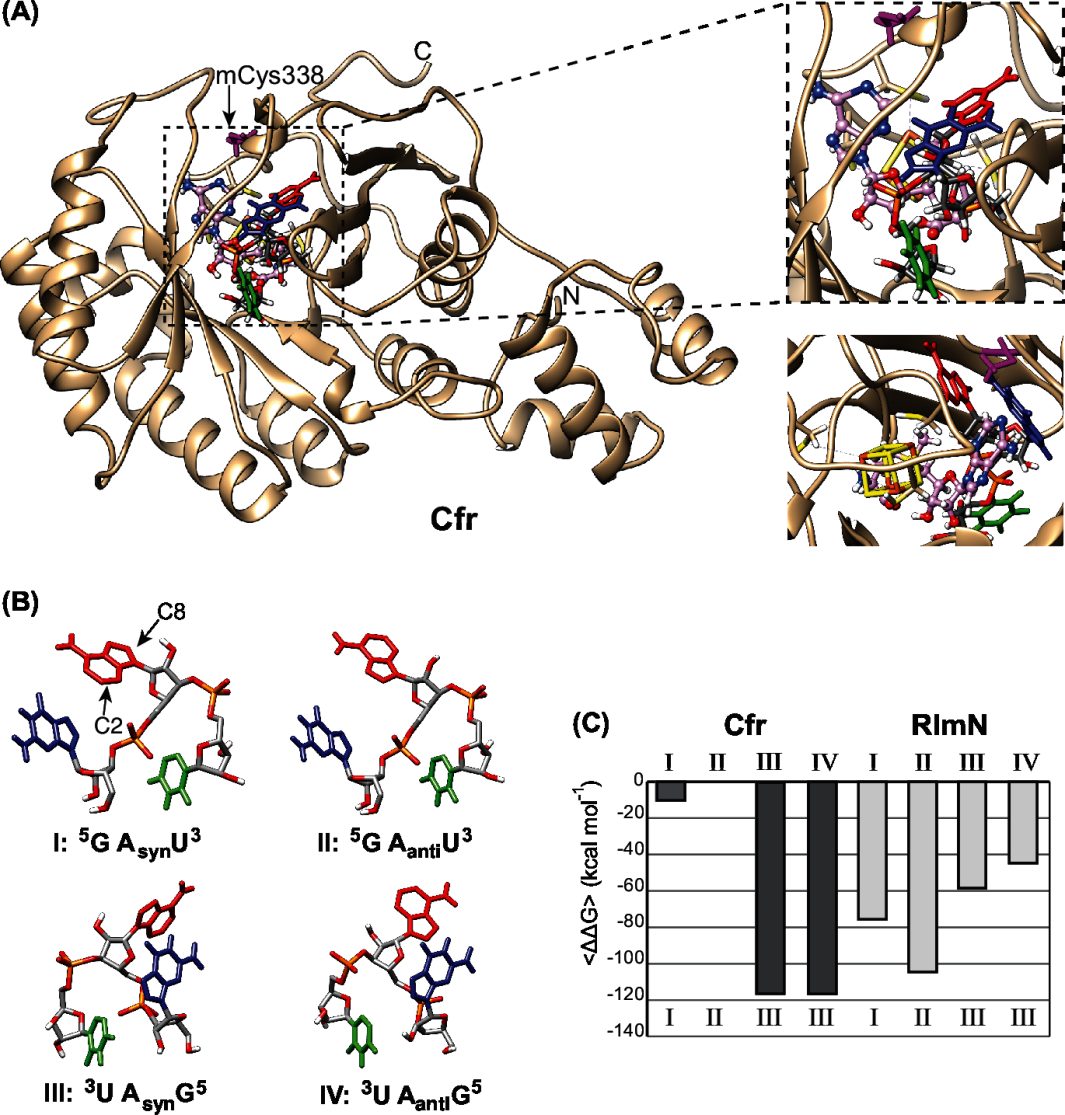
The Cfr structure model, the four starting poses of ligand GpApU and target binding affinities. (A) The whole Cfr model structure is shown at the left in tan with a trinucleotide target, a SAM molecule and a [4Fe-4S] cluster at the active site. The blow up at the right shows a detailed view of the active site with SAM in pink ball and sticks, the [4Fe-4S] cluster in yellow sticks and GpApU in sticks colored as in (B). The mCys338 is shown in purple. The blow up below corresponds to an approximately 120° turn of the active site. (B) Illustration of the four starting poses used for the MDs with guanine in blue, adenine in red and uracil in green and their labels used in the text below. (C) The data of the average binding energy of the trinucleotide ligand binding to Cfr or RlmN presented as relative differences. Each column represents the <ΔΔ*G>* data from ten snapshots of a trajectory. The MDs started with the poses corresponding to the numbers above the columns while the resulting poses of the ligands are depicted below the columns.

A Cfr homology model (I-TASSER server [29]) was produced using the RlmN X-ray crystal structure as a template similar to the presentation by Boal *et al*. [26]. The homology model is pictured in Fig 2A with the [4Fe-4S] cluster and a SAM molecule positioned in the same way as seen in the RlmN X-ray structure [26]. This was achieved by superimposing the two structures and transferring the cluster from the RlmN X-ray structure to the Cfr model. As mentioned in the introduction the methylation mechanism involves a transitory methylation of Cys338 for Cfr and Cys355 for RlmN and these were used as guiding amino acids for positioning the ligands. To make the two enzyme conformations as similar as possible, and because Cfr and RlmN are methylated before transfer of the methyl group to the substrate [26], a methyl group was also manually added to residue cysteine 338 of Cfr.

The investigation of target binding to the two enzymes started by placing a small target analogue, adenosine monophosphate (AMP), in the active site. Both Cfr and RlmN were able to accommodate the AMP ligand demonstrating ample space for larger ligands. This was followed by binding a trinucleotide ligand (GpApU) with the sequence corresponding to positions 2502–2504 of the 23S rRNA of *E*. *coli*. Since MD will not explore major changes in configurations, the ligand was placed in four distinct starting poses in each enzyme. The poses consist of two orientations of the ligand; one termed ^5^GAU^3^ and the other, a 180° turn, termed ^3^UAG^5^, and then the syn and anti conformations of the target adenine nucleobase. The four starting poses (Fig 2B) are named I: ^5^GA_syn_U^3^, II: ^5^GA_anti_U^3^, III: ^3^UA_syn_G^5^, IV: ^3^UA_anti_G^5^ to characterize their differences in direction and base conformation although they are all the same molecule. Both enzymes accommodate the ligand in their catalytic sites, as shown by negative values of the predicted Glide Scores, with values varying from -28.31 to -67.10 kcal mol^-1^.

After calculating the Glide Score, to ensure accommodation of the ligand, the MDs were conducted with the four poses I-IV of the trinucleotide ligand. The MD calculations for Cfr were conducted both with and without an added methyl group to residue Cys338 to analyse if this change had a significant effect on ligand accomodation, and it did not. As the RlmN X-ray structure contains the corresponding methylation, the mCys338 was chosen for the comparison. Ten snapshots were saved from each trajectory for each pose. The free energy of binding (Δ*G*) was calculated by the MM/GBSA method for each of the snapshots as well as the average of the Δ*G* values (<Δ*G*>) for each trajectory. The entropy term (TΔ*S*) of the model system was not ascertained, but is considered to stay relatively unchanged from one simulation to another, as the ligand is the same. For that reason, the comparison of the MD results was conducted using the average of the ΔΔ*G* values (<ΔΔ*G*>). The <ΔΔ*G*> values (Fig 2C) reflect the relative differences in binding affinity of the various poses with the trajectory possessing the smallest averaged affinity as reference point. The large spread in the <ΔΔ*G*> values from 10.29 kcal mol^-1^ up to 116.60 kcal mol^-1^, clearly show that the initial pose of the ligand affects the strength of the final binding configuration. The relative position of the ligand was visually assessed after the MDs to evaluate major structural changes and only poses similar to I, II and III were observed in both Cfr and RlmN. Thus, the adenosine conformation in pose IV, in both enzymes, turned from anti to syn during the MD. For Cfr the conformation III resulting from both initial poses III and IV, possessed almost the same binding affinities with <ΔΔ*G*> values of -116.14 and -116.60 kcal mol^-1^ respectively, whereas for RlmN an apparent conformation III resulting from initial poses III and IV showed different binding affinities, namely -58.42 and -44.69 kcal mol^-1^. The difference is expected to be due to small differences in the relative placement of the non-adenine part of the ligand in RlmN. In the Cfr model the amino acids from Ile334 to Ala337 form a 3_10_-helix prior to the MDs. Examining the Cfr model after the MDs, this helix is not present anymore with the ligand in the ^5^GAU^3^ orientation whereas the 3_10_-helix is maintained with the ligand in the ^3^UAG^5^ orientation. Opening the helix may induce a cost consistent with the lower affinity for the ^5^GAU^3^ orientations.

According to the data in Fig 2C, Cfr shows a clear preference for pose III with a high binding energy and rejects pose IV by turning the adenine nucleobase. In contrast the RlmN structure shows a higher variation in its binding modes with a preference for ^5^GAU^3^, but also the ability to turn the adenine nucleobase from anti to syn in the ^3^UAG^5^ orientation. The data points to a major difference between the enzymes with Cfr showing a preference for the ^3^UAG^5^ orientation whereas RlmN prefers ^5^GAU^3^ indicating an approximately 180° turn in the way the enzymes target the RNA and thus a different mode of binding. We do not know if this reflects a true difference in the function of the enzymes or if there could be other elements outside the catalytic crevice that strongly influence the orientation of the RNA binding.

While Cfr shows one preferred pose, RlmN shows less energy differences between the three poses. In contrast to the hypothesis, it appears as if RlmN is more flexible in target binding than Cfr, regardless of the fact that Cfr has the ability to modify both C2 and C8. The flexibility in target binding for RlmN could reflect its ability to use other targets as A37 in some tRNAs that have already been shown to be modified by RlmN [22]. An explanation for Cfr could be that it primarily has to methylate C8 in pose III and that this will then change the affinity so as to place C2 for an additional methylation.

The computational approach has its limitations as we are dealing with a complicated reaction mechanism involving transient protein methylation, two SAM molecules and transient protein-RNA crosslinking. Our calculations are based on the available static X-ray structure of RlmN and we cannot exclude structural rearrangement that might affect the target binding. Having said that, it is still possible to extract useful information from the models. The docking shows that there is ample space in the funnel of the catalytic site in both enzymes for binding of the trinucleotide ligand in various ways. The Cfr model shows a strong preference for one target pose while RlmN appears more flexible. Additionally, there is no clear sign of steric hindrance that could explain why RlmN methylates only C2 and Cfr preferentially C8 but also C2. Most surprising is the indication of a 180° difference in positioning of the target which points to a major evolutionary differentiation of the two enzymes despite their similarities.

### Defining regions of the active site to be interchanged to investigate C8/C2 specificity

**Fig 3:**
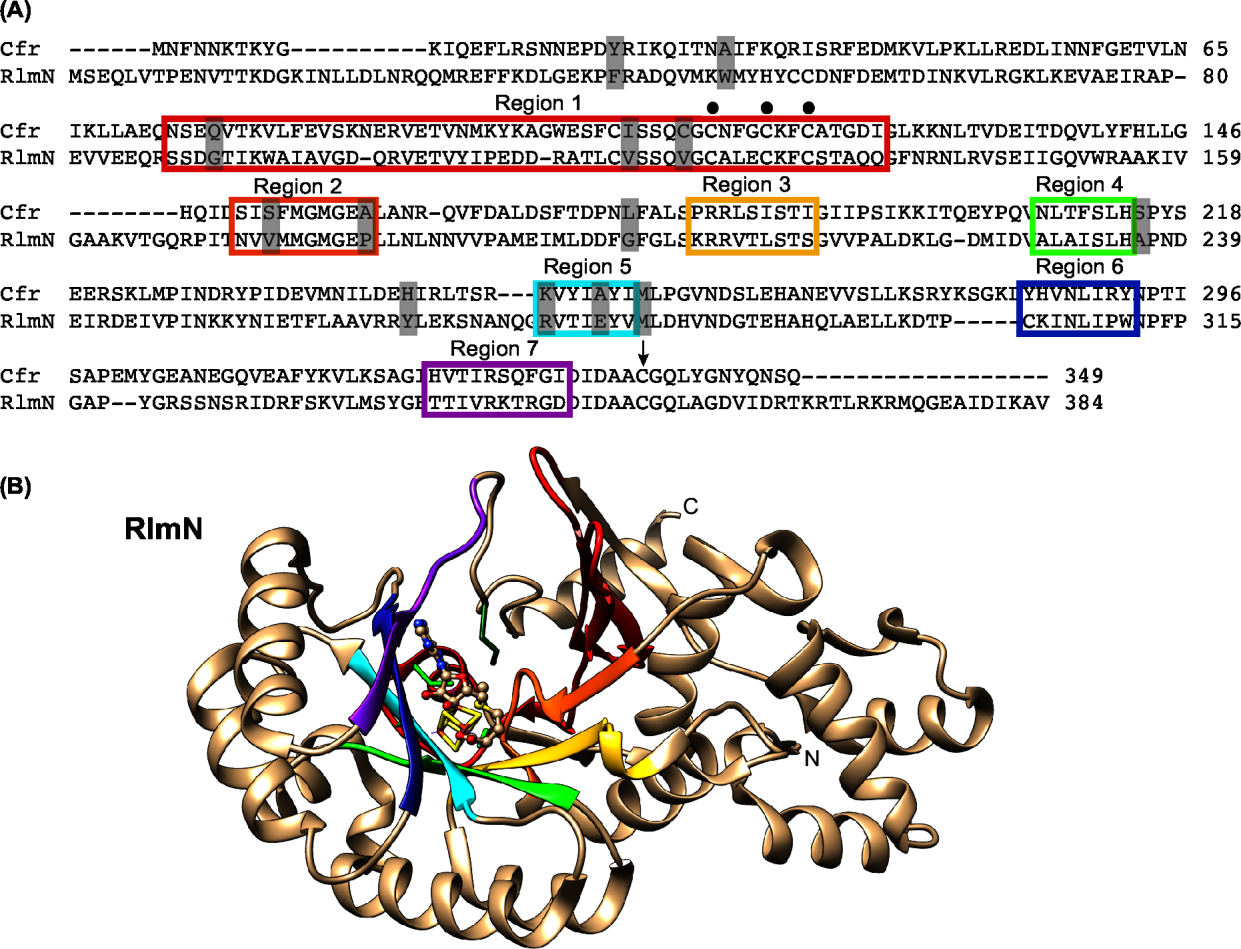
The interchanged regions for the investigation of C2/C8 specificity. (A) Alignment of the amino acid sequences of Cfr (GI: 34328031 / NCBI: NP_899167.1) and RlmN (GI: 16130442 / NCBI: NP_417012.1). The seven coloured boxes depict the regions that encompass the active site of the enzymes. The dots above the sequence indicate the CX_3_CX_2_C motif. The black arrow shows the cysteine 338/355. The grey shading mark the 13 selectively conserved positions in Cfr- or RlmN-like proteins. (B) Representation of the X-ray crystal structure of RlmN (PDB file 3RFA) [26] with the same region colouring as in the amino acid alignment and oriented similar to the Cfr model structure in Fig 2A. Also, the SAM molecule and the [4Fe-4S] cluster are included as in Fig 1A. The three cysteines in the CX_3_CX_2_C as well as the Cys355 are shown in green sticks.

The sequences of the Cfr (349 aa) and *E*. *coli* RlmN (384 aa) enzymes are very similar sharing 57 strongly conserved amino acids [15]. The alignment of Cfr and *E*. *coli* RlmN is illustrated in Fig 3A and have 30% identity and 46% similarity. Thirteen amino acids have been shown to be selectively conserved for each class, meaning that >70% of all RlmN-like sequences have a specific amino acid and >70% of Cfr-like sequences have another specific amino acid in the same positions [15]. Using the RlmN X-ray crystal structure and defining the active site as where the SAM and [4Fe-4S] cluster bind, and where the enzyme has a methylated Cys355 for donating the methyl group to A2503, we have selected the seven regions shown as colored beta sheets in Fig 3B. The same regions are marked as colored boxes in the alignment (Fig 3A). They constitute the central domain of the enzymes, forming an incomplete TIM-barrel that is characteristic for radical-SAM enzymes [37]. These regions also contain about half of the selectively conserved amino acids (grey shading in Fig 3A). The corresponding regions in the Cfr homology model are positioned very similarly to the ones seen in the RlmN structure. From the docking presented above we expect the specificity regarding C8 or C2 methylation of A2503 to reside somewhere in these seven regions as they constitute almost the entire groove. The C8/C2 specificity was therefore investigated by exchange of the corresponding gene regions in *cfr* and *rlmN*, expression of the mixed genes from plasmids and testing which of them would alter the specificity from C8 to C2 or vice versa or show the dual specificity.

### Construction and expression of mixed genes to analyse Cfr and RlmN specificities

To have a suitable constitutive expression of genes for comparison of the function of the mixed enzymes, we substituted the coding region of the *tet* gene of pBR322 with the genes to be investigated in this study. We inserted a synthetic version of the *cfr* gene to get an *E*. *coli* optimized codon usage, and obtained plasmid pBRCfr. The functionality of the enzyme was ensured by an appropriate antibiotic resistance pattern. In pBRRlmN the *E*. *coli rlmN* replaced the *tet* gene of pBR322. With these two plasmids as a basis we created a variety of different mixed genes replacing selected regions from one enzyme with counterpart regions from the other enzyme. The construction of the plasmids using restriction enzyme digests and/or overlap extension PCR is explained in the Materials and Methods. The mixed genes contained from one to seven regions replaced from the corresponding regions of the other gene. The regions contain from three to 31 amino acid replacements and two amino acid deletions as can be seen in Fig 3A. Regions 1, 2 and 5 contain selectively conserved amino acids while the others do not. The plasmid constructs are listed in Table 1 along with additional information of relevant features of the cloned genes. The numbers in the plasmid names refer to the exchange of the regions shown in Fig 3. The plasmids were transformed into *E*. *coli* JW2501-1 for RNA primer extension analysis and into *E*. *coli* AS19 for MIC determination.

**Table 1:** Summary of plasmid constructs and primer extension results from *E*. *coli* JW2501-1 harbouring the plasmids.

Plasmid	A2503 stop	Remarks on gene constructs
pBRCfr	+++	codon optimized *cfr* gene
pBRCfrRlmN	-	approximately half *cfr* gene + half *rlmN* gene
pBRRlmNCfr	-	approximately half *rlmN* gene + half *cfr* gene
pBRCfr1234567rRlmN	-	*cfr* gene with regions 1–7 of *rlmN* gene
pBRCfr34567rRlmN	-	*cfr* gene with regions 3–7 of *rlmN* gen
pBRCfr12rRlmN	-	*cfr* gene with regions 1 and 2 of *rlmN* gene
pBRCfr234567rRlmN	-	*cfr* gene with regions 2–7 of *rlmN* gene
pBRCfr1XrRlmN	-	*cfr* gene with region 1 of *rlmN* gene
pBRCfr2XrRlmN	(+)	*cfr* gene with region 2 of *rlmN* gene
pBRCfr3XrRlmN	++	*cfr* gene with region 3 of *rlmN* gene
pBRCfr4XrRlmN	+	*cfr* gene with region 4 of *rlmN* gene
pBRCfr5XrRlmN	-	*cfr* gene with region 5 of *rlmN* gene
pBRCfr6XrRlmN	-	*cfr* gene with region 6 of *rlmN* gene
pBRCfr7XrRlmN	(+)	*cfr* gene with region 7 of *rlmN* gene
pBRRlmN1234567rCfr	-	*rlmN* gene with regions 1–7 of *cfr* gene
pBRRlmN34567rCfr	-	*rlmN* gene with regions 3–7 of *cfr* gene
pBRRlmN12rCfr	-	*rlmN* gene with regions 1 and 2 of *cfr* gene
pBRRlmN	+	*rlmN gene*
pBR322	-	Parent plasmid
No plasmid	-	Host control

The strength of the modification stop on gels from the primer extension was visually assessed and is indicated by 1–3 plusses. (+) Ambiguous band, - no band present.

### Modification at A2503 23S rRNA as assay of enzyme function

Reverse transcriptase pauses or stops at several kinds of RNA modifications including methylation of C2 and C8 [19, 38]. Methylation of C8 gives rise to a strong stop while methylation of C2 only causes a weak stop band, and cannot be distinguished from a partial methylation of C8. Cfr also represses the ribose methylation of C2498 of the 23S rRNA [18] whereas RlmN does not demonstrate the same effect. As RlmN is naturally present in *E*. *coli* we used a knock out strain with a non-functional *rlmN* gene (*E*. *coli* JW2501-1) [33] as host for the plasmids to investigate the RNA methylation at A2503 in 23S rRNA, as described in our previous study [19]. Total RNA was isolated from strains with the plasmids from Table 1 and primer extension analysis of the region around A2503 was performed followed by gel electrophoresis.

**Fig 4:**
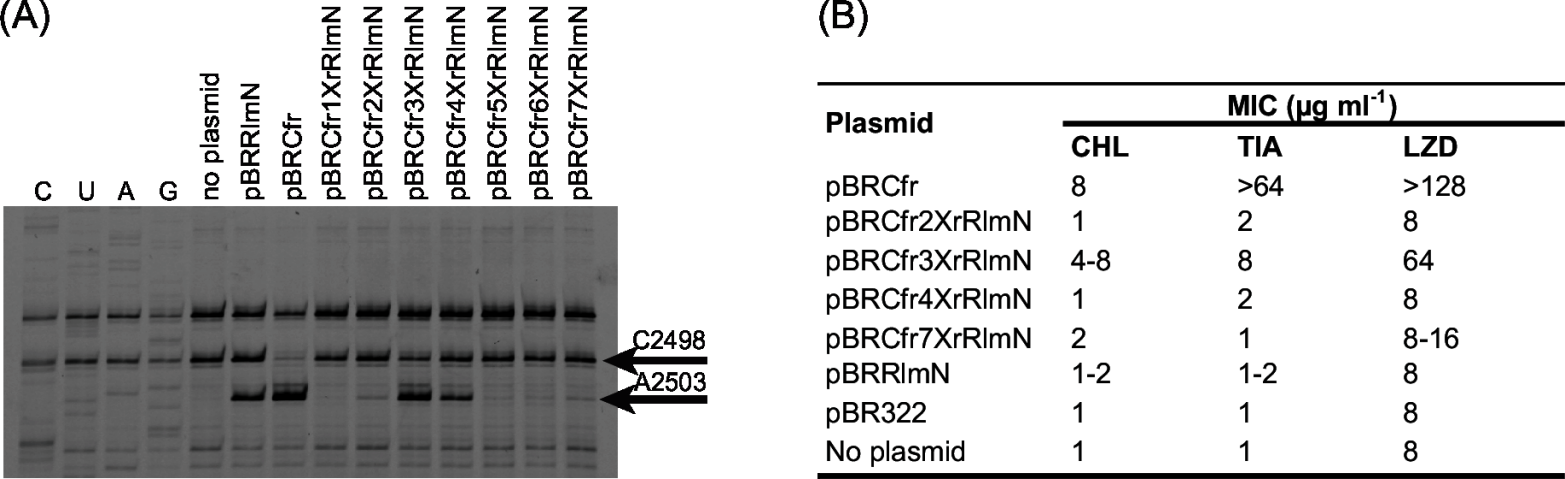
Primer extension analysis and MICs. (A) A gel picture with a selection of primer extension results is shown to illustrate the A2503 23S RNA modification stop. The 23S RNA are from *E*.*coli* JW2501-1 strains harbouring the plasmids listed above the gel lanes. The region shown is limited to the nucleotides flanking A2503 that is methylated by Cfr and C2498 where Cfr inhibits methylation (indicated by arrows). Lanes 1–4, marked C, U, A, and G, refer to dideoxynucleotide sequencing reactions. Reverse transcriptase stops one nucleotide before the corresponding nucleotide in the sequencing lanes. (B) The MICs of *E*. *coli* AS19 strains harbouring the plasmids providing a modification stop with the primer extension analysis and controls. The numbers are the average of at least three independent experiments. An interval is given when no clear distinction between the values was obtained and only >2-fold differences are considered significant.

A selection of samples including those from Cfr and RlmN and controls are presented in Fig 4A showing the A2503 modification stop together with other nearby stops caused by either other modifications, structural hindrance of extension or hydrolysis. A strong band appears in presence of Cfr and a modest band in presence of RlmN. The strength of all bands was visually assessed as indicated by the plusses in Table 1.

Surprisingly, very few of the mixed genes appear to give rise to functional enzymes. Only one of the mixed constructs caused a relatively strong stop namely from pBRCfr3XrRlmN plasmid that contains a 5 amino acid replacement in region 3 from Cfr to the RlmN counterpart. Some activity is provided by pBRCfr4XrRlmN that has just 3 amino acids exchanged in region 4 of Cfr to the RlmN counterpart. Finally, a minor effect was discernible in the lanes containing RNA from strains JW2501-1/pBRCfr2XrRlmN and JW2501-1/pBRCfr7XrRlmN but it is uncertain if this is due to a true minor modification effect or artifact of the primer extension method. The rest of the constructs do not confer any stop-band and apparently do not provide any A2503 modification even though some of them only consist of relatively small exchanges of the two similar enzymes. For pBRCfr5XrRlmN and pBRCfr6XrRlmN the changes are only 4 and 5 amino acid replacements, but still they abolish entirely the activity. Unfortunately, there is no distinct correlation between the effect of replacement with regions that contain or not selectively conserved amino acids, which could have pointed to the site of specificity. The lack of function of the replacements is likely due to the change of functionally important amino acids indicating that Cfr and RlmN have a high individual specificity despite their similarities. Lack of modification could also be due to instability of the enzyme or other problems with expression. Even though this is an interesting question it is not considered relevant to this study as the focus is on the difference in C2/C8 A2503 specificity. Replacing the whole active site by creating pBRCfr1234567rRlmN and pBRRlmN1234567rCfr also completely prevents activity of the enzymes. The same applies for the constructs containing a ratio of approximately half and half of each enzyme, which is maybe not surprising knowing the results for the individual regions.

### Antibiotic resistance as assay for the m^8^A2503 modification

Cfr is known to confer resistance to six distinct classes of antibiotics by m^8^A2503 methylation [4–7] while RlmN does not. We can thus use antibiotic resistance to distinguish whether a stop in the primer extension assay at A2503 is due to an m^2^A2503 modification by an RlmN-like enzyme or to an m^8^A2503 by a Cfr-like enzyme. In order to investigate if strains hosting the mixed enzymes, and showing an A2503 stop in 23S rRNA, confer resistance we determined MICs for these strains. We chose chloramphenicol, tiamulin and linezolid as three antibiotics to represent the Cfr resistance effect. The MICs were determined with the broth microdilution method for *E*. *coli* AS19 with the plasmids that provided even a weak primer extension stop. The *E*. *coli* AS19 is used because it is more sensitive to many antibiotics compared to other *E*. *coli* strains that have a naturally low sensitivity level. The MICs are presented in Fig 4B and show resistance for the *E*. *coli* AS19/pBRCfr expressing Cfr as expected. Also as expected *E*. *coli* AS19/pBRRlmN does not confer resistance and shows MICs of the same magnitude as the strain without a plasmid. As indicated from the primer extension, pBRCfr2XrRlmN and pBRCfr7XrRlmN showed also no reduced antibiotic sensitivity. The enzyme encoded by plasmid pBRCfr3XrRlmN shows a reduced susceptibility to all three antibiotics, although the effect is smaller than that caused by pBRCfr. Thus, the Cfr3XrRlmN mixed enzyme has a decreased function compared to the Cfr enzyme. Region 3 with 11 amino acids, where 5 of them have been exchanged to the RlmN counterpart, is positioned in close proximity to the carboxyl group of the SAM and the [4Fe-4S] cluster. The strain with pBRCfr4XrRlmN does not show any lowered sensitivity to the antibiotics although pBRCfr4XrRlmN provided a faint but clear primer extension stop. The weak band could represent either a very low Cfr-like activity that is too small to be reflected in the MICs or a low RlmN activity although we find this highly unlikely as none of the other constructs showed similar behaviour.

## Conclusions

By employing two distinct approaches we have shown that Cfr and RlmN are distant relatives, despite their sequence similarity and their shared target and mechanism. The computational approach, although with some limitations, provides useful information concerning the accommodation of the ligand in the active site of the enzymes and depicts differences in enzyme preferences. Especially, the indication of a target turn-around of approximately 180° depicts a major difference between the two enzymes. In the genetic approach with exchanges of regions of Cfr and RlmN, even only by exchanging a few nucleotides, the function of the enzymes is lost and demonstrates the evolutionary changes that Cfr and RlmN have undergone. RlmN has previously been shown to modify tRNA [22] and this could point to the possibility of Cfr also holding an alternate function. We suggest that both enzymes have been through long independent evolutions whereby adaptions and random changes have diversified their original homology and build up substantial differences. The study emphasises the difficulties in predicting enzyme function from sequence similarities and target identity.

## Supporting Information

S1 PDB File Cfr homology model used in the study. The model was generated as specified in the “Molecular dynamics simulations and calculation of the binding free energy” section.
(PDB)

S2 PDB File Cfr homology model containing the target trinucleotide, SAM, [4Fe-4S] cluster and mCys338. (PDB)
